# Cloning, Expression and Characterization of a Gene from Earthworm *Eisenia fetida* Encoding a Blood-Clot Dissolving Protein

**DOI:** 10.1371/journal.pone.0053110

**Published:** 2012-12-27

**Authors:** GangQiang Li, Kevin Yueju Wang, DaHui Li, Nan Wang, DeHu Liu

**Affiliations:** 1 Biotechnology Research Institute, Chinese Academy of Agricultural Sciences, Beijing, China; 2 Department of Natural Sciences, Northeastern State University, Broken Arrow, Oklahoma, United States of America; 3 Pharmaceutical School, Peking University, Beijing, China; Rush University Medical Center, United States of America

## Abstract

A lumbrokinase gene encoding a blood-clot dissolving protein was cloned from earthworm (*Eisenia fetida*) by RT-PCR amplification. The gene designated as CST1 (GenBank No. AY840996) was sequence analyzed. The cDNA consists of 888 bp with an open reading frame of 729 bp, which encodes 242 amino acid residues. Multiple sequence alignments revealed that CST1 shares similarities and conserved amino acids with other reported lumbrokinases. The amino acid sequence of CST1 exhibits structural features similar to those found in other serine proteases, including human tissue-type (tPA), urokinase (uPA), and vampire bat (DSPAα1) plasminogen activators. CST1 has a conserved catalytic triad, found in the active sites of protease enzymes, which are important residues involved in polypeptide catalysis. CST1 was expressed as inclusion bodies in *Escherichia coli* BL21(DE3). The molecular mass of recombinant CST1 (rCST) was 25 kDa as estimated by SDS–PAGE, and further confirmed by Western Blot analysis. His-tagged rCST1 was purified and renatured using nickel-chelating resin with a recovery rate of 50% and a purity of 95%. The purified, renatured rCST1 showed fibrinolytic activity evaluated by both a fibrin plate and a blood clot lysis assay. rCST1 degraded fibrin on the fibrin plate. A significant percentage (65.7%) of blood clot lysis was observed when blood clot was treated with 80 mg/mL of rCST1 *in vitro*. The antithrombotic activity of rCST1 was 912 units/mg calculated by comparison with the activity of a lumbrokinase standard. These findings indicate that rCST1 has potential as a potent blood-clot treatment. Therefore, the expression and purification of a single lumbrokinase represents an important improvement in the use of lumbrokinases.

## Introduction

Lumbrokinases are a group of proteases with molecular weights in the range of 25 to 32 kDa [1]. They are present in the body cavity, intestine tissue, tissue fluid and intestinal fluid [2] of earthworms (*Eisenia fetida*) and have been studied as a thrombolytic drug for various clinical conditions, including acute, sub-acute, and chronic diseases associated with thrombosis [3]–[10]. Commercial lumbrokinase products have been administered in Asia and North America (Boluoke®) to treat hypercoagulation. Lumbrokinases can directly dissolve fibrinogen and fibrin [6]–[13], convert plasminogen to plasmin, and increase endogenous human tissue plasminogen activator (t-PA) activity to dissolve fibrin clots. Studies have shown that lumbrokinases inhibit platelet activation and aggregation and blocks the intrinsic coagulation pathway [11], [12]. Unlike t-PA, lumbrokinases exhibit thrombolytic activity only in the presence of fibrin. Therefore, lumbrokinase has the advantage of not causing excessive bleeding [14], [15].

Lumbrokinase is safe, non-toxic, and has few side effects [3], [6], [7], [15], [16]. It can be given orally, which is very convenient for patient use [16], [17]. In China and other parts of the Far East, oral administration of earthworm powder has been used widely as a drug for the prevention and treatment of various diseases for several thousand years. Clinical studies indicate that orally-administered lumbrokinase is very effective in reducing coagulation of fibrin and blood platelets, and has no obvious side effects on nervous or respiratory system function, blood vessels, liver, or kidneys [6], [7].

Traditional extraction and purification of lumbrokinase from earthworms is complex and the yield is relatively low. Current lumbrokinase products contain many different earthworm fibrinolytic enzymes (EFEs), and a number of contaminants which can induce nausea and vomiting [18]. Therefore, the identification and expression of a single recombinant lumbrokinase via genetic engineering is urgently needed.

Earthworm species, such as *Lumbricus rubellus*, *Eisenia fetida* and *Lumbricus bimastus*, are the most common source of lumbrokinase. Although over 15 cDNAs of lumbrokinases have been reported in NCBI GenBank, very few have exhibited fibrinolytic activity when cloned and expressed in *E. coli* [11], [19], *Pichia pastoris* [20], [21] or goat mammary glands [18]. In general, the expressed lumbrokinases had either a low yield or only exhibited partial activity. In the present study, we cloned a lumbrokinase cDNA from earthworm, *E. fetida*. The new fibrinolytic enzyme gene, designated as *cst1* (GenBank: AY840996), was expressed in *E. coli,* recovered, further purified with a nickel-chelating resin column, and evaluated for activity with a fibrin plate assay. Results showed that recombinant CST1 (rCST1) has strong fibrinolytic activity. Blood clot lysis was observed when rCST1 was tested *in vitro*. The results may lead to the development of a therapeutic thrombotic agent for the treatment of diseases associated with thrombosis.

## Materials and Methods

### Materials

Earthworm (*E.fetida*) served as the source for obtaining lumbrokinase total RNA. Earthworms were kindly provided by Dr. Zhengji Yang, Institute of Natural Resources and Regional Planning, Chinese Academy of Agricultural Science (CAAS), Beijing, China. Kunming white mice were purchased from the Medical School of Peking University, China. Mice usage protocol was supported by CAAS Institutional Ethics Committee. The standard lumbrokinase product (1200 U/mg) was purchased from Baiao Pharmaceuticals Co., LTD (Beijing, China).

### Total RNA isolation, cDNA synthesis, cloning and sequence analysis

Total RNA was extracted from a fresh earthworm (0.89g) with a Flash UNIQ-10 RNA extraction kit (Shanghai Biotechnology Company, China) according to the manufacturer's protocol. DNase I was used to exclude the possibility of chromosomal DNA. The presence of RNA was confirmed by 1% agarose gel electrophoresis. cDNA was prepared using RNA LA PCR™ reverse transcriptase Kit (TaKaRa, Dalian, China). Two µg of total RNA were mixed with 1 µl reverse transcriptase (200 U/µl) and incubated for 5 min at 70°C, reverse transcribed for 1 h at 42°C, and inactivated for 5 min at 95°C. The oligonucleotide primer sequences used for *CST1* were forward primer-PL1: 5′-GAGAATTCGTGATAGGGGGCACTAACGCTA-3′ and reverse primer-PL2: 5′-CGCAAGCTTCTATGAACGGATTTAATGAGG-3′ (synthesized by TaKaRa, Dalian, China). EcoRI and HindIII restriction sites are underlined. PL1 and PL2 were designed using the sequence of *L. bimastus* fibrinolytic enzyme mRNA (GenBank: AF109648). PCR amplification was conducted using High Fidelity *Taq* DNA Polymerase (TaKaRa Biotech, Dailan, China) using the following program: denaturation at 94°C for 4 min, followed by 35 cycles of melting at 92°C for 30 sec, annealing at 55°C for 45 s, and extension at 72°C for 90 min. The resulting PCR products were visualized on a 1% agarose gel. The purified DNA fragment was ligated into a TA cloning vector, pMD18-T, and transformed into DH5α competent bacterial cells. The resulting plasmid, pMD/CST1, was sequenced by TaKaRa (Dalian, China). EnzymX 3 software (http://enzymex.en.softonic.com/mac) was used to identify the CST1 cDNA open reading frame. DNA translation was performed with DNAtoPROTEIN software (http://www.dnatoprotein.com; A. Boto, John Hopkins School of Medicine). Sequence analysis, multiple sequence alignment, and phylogenetic analysis were conducted with ClustalW2 (http://www.ebi.ac.uk/Tools/msa/clustalw2/) software.

### Expression and purification of recombinant lumbrokinase

After sequencing, the CST1 fragment was isolated from pMD/CST1 with EcoRI/HindIII and cloned into the, pET28a(+) His-Tag (Novagen) bacterial expression vector, yielding pMD/CST1. Plasmid pMD/CST1 was transformed into the expression strain of *E. coli,* BL21(DE3), and the resulting culture was spread out on LB plates containing selective antibiotics (Kanamycin 50 mg/L and Chloramphenicol 34 mg/L). A single colony was inoculated into 5 mL of LB medium containing Kanamycin (50 mg/L) and grown overnight at 37°C, 250 rpm. One mL of the culture was then used to inoculate 100 mL of fresh LB medium. The culture was grown at 37°C, 250 rpm until the OD_600_ reached 0.6 after which the culture was supplemented with 1.0 mM isopropyl-β-D-thiogalactopyranosid (IPTG). After 6 h of induction, a 2 mL sample was pelleted by centrifugation and re-suspended in 1×SDS loading buffer and boiled for 3 min. The supernatant was collected after centrifugation at 6500 g for 10 min and run on 12% SDS-PAGE for protein analysis. The protein in the remainder of the culture was collected by centrifugation and the pellet was resuspended in lysis buffer (0.05 M Tris-HCl, pH8.0, 1 mM EDTA, pH 8.0, 0.1 M NaCl, and 2 mg/mL lysozyme). The cells were then homogenized by sonication and the supernatant was collected after centrifugation (13000 g, 15 min) at 4°C. The insoluble fraction was washed three times with 30 mL of inclusion body wash buffer (0.05 M Tris-HCl buffer, 10 mM EDTA,0.5% TritonX-100, pH8.0) and resuspended in 30 mL of inclusion body lysis buffer (0.05 M Tris-HCl buffer, 1 M PMSF, 8 M urea and 10 mM DTT, pH8.0). The suspension was left at room temperature undisturbed for 4 h and then centrifuged at 12,000 g for 15 min. 5 µL of the collected lysate was analyzed by 12% SDS-PAGE.

Recombinant CST1 was recovered by dialysis. Supernatant that was previously collected was dialyzed against the recovery buffer (0.05 M Tris-HCl, 6 M urea, 2 mM glutathione (GSH) and 1 mM oxidized glutathione (GSSG, pH8.0) using a 12,000–14,000 MWCO membrane (GreenBird Co., Ltd, Shanghai, China) at 4°C for 4 h. The recombinant protein was then further dialyzed at 4°C for 2 h against the recovery buffer containing various concentrations of urea (4, 3, 2, 1 and 0.5 M), pooled and stored at 4°C overnight. Dialysis buffer (0.05 M Tris-HCl buffer, 2 M GSH, 1 M GSSG, pH8.0) was then used to remove the excess urea.

For purification of poly-His:CST1 recombinant protein, the 10,000 g (15 min) supernatant was loaded on a prepared purification ProBond™ column (a nickel-chelating resin) equilibrated in Native Binding Buffer as described by the manufacturer (Invitrogen Carlsbad, CA, USA). The supernatant was allowed to bind to the resin for 1 h using gentle agitation. The resin was settled by gravity and washed with 8 mL Native Wash Buffer (8 M Urea, 20 mM sodium phosphate, pH 6.0 and 0.5 mM NaCl) three times. The bound protein was eluted from the column with 10 mL Native Elution Buffer (8 M Urea, 20 mM NaH2PO4, pH 4.0 and 0.5 mM NaCl). Proteins were analyzed by 12% SDS-PAGE. Densitometry was performed to measure the relative amount of the expressed protein. Proteins on 12% SDS-PAGE gels were scanned and analyzed with LabWork 3.0 software (UVP LLC). Recombinant CST1 protein content was estimated by comparing the intensity of the stained band with that of bands containing a known amount of lumbrokinase standard on 12% SDS-PAGE gel. The eluted protein was frozen immediately at −80°C for 60 min, and freeze-dried at 15 Pa and −55°C for 24 h. The protein powder was re-suspended in 1×PBS for further analysis.

### CST1 antiserum preparation and antibody ELISA detection

Six 5–7 week old male Kunming white mice (purchased from Medical School of Peking University, China) were immunized subcutaneously with 50 µg of the rCST1 protein mixed with Freund's incomplete adjuvant (IFA) in a 1∶1 ratio. Two subsequent booster injections were given at two-week intervals with the same preparation of rCST1 protein. One week after the fourth immunization, blood samples were taken from the retro-orbital sinus and centrifuged. The antibody titer was determined in the obtained sera by ELISA analysis. Pre-immune serum was used as negative control. Samples were diluted to 5 µg/mL in pH 9.6, 0.05 M sodium bicarbonate solution, and then coated in wells of an ELISA plate and left to stand overnight at 4°C. The wells were washed with PBST buffer (1× Phosphate Buffered Saline Tween-20) three times and then sequentially incubated with anti-CST1 serum at serial dilutions (from 1/200, 1/400, 1/800 to 1/51200) for 2 h at 37°C. After three rinses with PBST, the wells were blocked with Blocking Buffer (5% Skim Milk Powder in 1×PBS) for 1 h at 37°C. Blocking Buffer was removed by flicking plate. The plate was washed three rinses with PBST and was incubated in a 1∶3000 dilution of alkaline phosphatase-conjugated Rabbit Anti-mouse IgG. p-Nitrophenyl Phosphate Disodium (pNPP) solution was used as a colorimetric reagent. The staining level was measured using an ELISA plate reader (UVP, LLC, Upland, California, USA). The titer cut-off value was determined to be 1/12800 by absorbance at OD405.

### Western blot analysis

Proteins (20 µg) from total crude lysate (with or without IPTG induction), purified rCST1, and a commercial lumbrokinase product were separated by 12% SDS-PAGE, and transferred to a nitrocellulose membrane with iBlot® blotting system (Invitrogen, U.S.A) respectively. Membranes were incubated with 3% bovine serum albumin (BSA) in TBST (25 mM Tris–HCl, pH 7.4, 0.14 mM NaCl, and 0.05% Tween 20) for 2 h at 4°C to block nonspecific binding. After rinsing three times with TBST, the blocked membrane was incubated with diluted (1∶3000) mouse anti-CST1 serum (prepared as previously described) overnight at 4°C. The membrane was rinsed thoroughly with PBST and then incubated with a 1∶5000 dilution of alkaline phosphatase-conjugated rabbit anti-mouse for 1 h at 37°C. The membrane was rinsed with PBST and bound antiserum was visualized with NBT/BCIP (nitro-blue tetrazolium/5-bromo-4-chloro-3′-indolyphosphate, Sigma-Aldrich Co. LLC, U.S.A).

### Fibrinolytic activity assay

Fibrinolytic enzyme activity was detected by a modified fibrin plate method [22], [23]. A 50 mL of 1.0% agarose in 1×PBS buffer was boiled in a 200 mL conical flask and left it to cool at 60°C water bath. 1 µg/mL of fibrinogen and 0.1 IU/mL of thrombin were added and swirled to mix. The mixture solution was slowly poured into the petri dish. The plate was left undisturbed until the agarose solidified. Wells (5 mm diameter) were formed in each plate with an aseptic hole punch. Fifty µL of various proteins samples (0.5 mg protein/mL) were loaded in each well and incubated at 37°C overnight. The activity of samples was calculated as the lytic area surrounding the well on the fibrin plate compared to standard lumbrokinase (1200 u/mg). The diameter of the clear area around each well was measured in mm with a ruler. The diameter of the well was subtracted from the measured diameter of each well. Mean values are an average of three replicates (n = 3).

### Blood-clot lysis activity assay

An *in vitro* mice blood-clot lysis activity assay was used as described by Prasad et al [24]. Fresh mouse blood was drawn from the retro-orbital sinus. 600 µL of blood was transferred to pre-weighed 1.5 mL Eppendorf tubes and incubated at 37°C for 2 h. Serum was completely removed after clot formation. The tubes containing the clots were then weighed again. Clot weight was determined by subtracting the weight of each tube (clot weight = weight of clot containing tube – empty tube weight). 200 µL of lumbrokinase (1200 U/mg) or various concentrations (5, 10, 20, 40 and 80 mg/mL) rCST1 dissolved in 1×PBS was added to the tubes containing clots. Unrefolded rCST1 proteins were also tested as negative control. Treated samples were incubated at 37°C overnight. Lysed fluid was completely absorbed from each tube with filter paper and the tubes were then re-weighed. Weight differences of each tube before and after incubation were calculated and the percentage of clot lysis in treated and untreated samples was recorded. Mean values are an average of three replicates (n = 3). Values are means ± SD (n = 3). The significant differences (*p*≤0.01) between means were evaluated by *t*-test.

## Results

### cDNA cloning and sequence analysis

**Figure 1:**
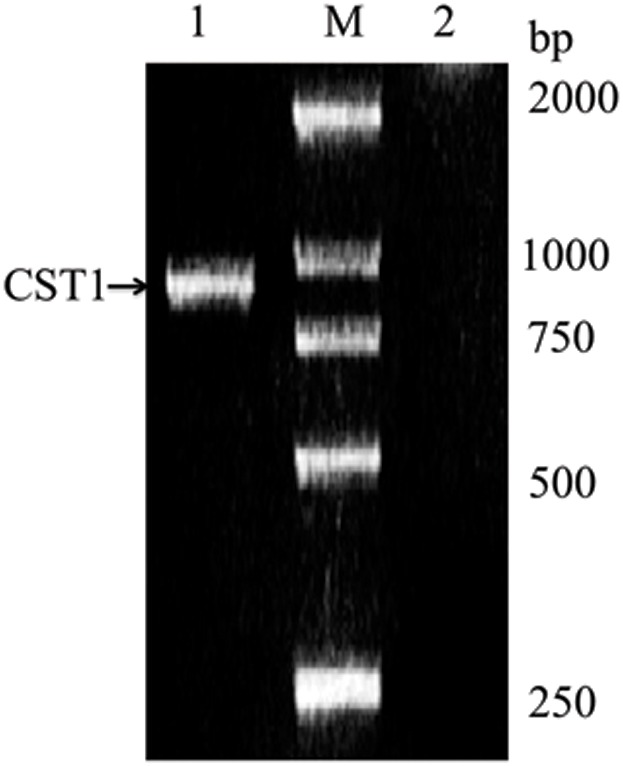
cDNA fragment of CST1. cDNA was obtained by RT-PCR amplification from earthworm, *E. fetida*, with specific primers designed according to the reported fibrinolytic enzyme mRNA (GenBank: AF109648.1) of *L. bimastus.* Lane 1: cDNA of CST1 was 888 pb in length; Lane M: molecular markers; Lane 2: Negative control was PCR amplification (without reverse transcriptase reaction) from total RNA extracted from *E. fetida.*

**Figure 2:**
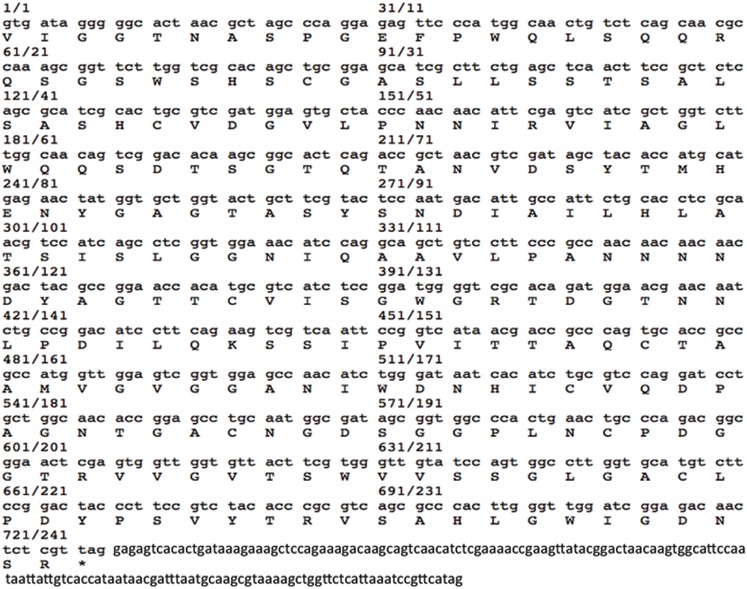
CST1 nucleotide (lower case) and deduced amino acid (upper case) sequences. A stop codon (tag) is indicated by an asterisk (*) followed by 159 nt non-translational region. GenBank accession no. AY840996.

The full length cDNA fragment of *cst1* was generated by RT-PCR using *E. fetida* total RNA as a template (Fig. 1). The nucleotide sequence and deduced amino acid sequence of CST1 have been deposited in GenBank (Fig. 2). The CST1 cDNA contains 888 nucleotides in length with a 159 nucleotide 3′UTR. The deduced amino acid sequence analysis exists within the open reading frame from 1 to 729 nt encoding a 242-aa protein with a theoretical isoelectric point (pI) of 4.85 and molecular weight (Mw) of 24.78 kDa (GenBank: AY840996.1; Protein ID: AAW27919.1).

### Homology and phylogenetic analysis

**Figure 3:**
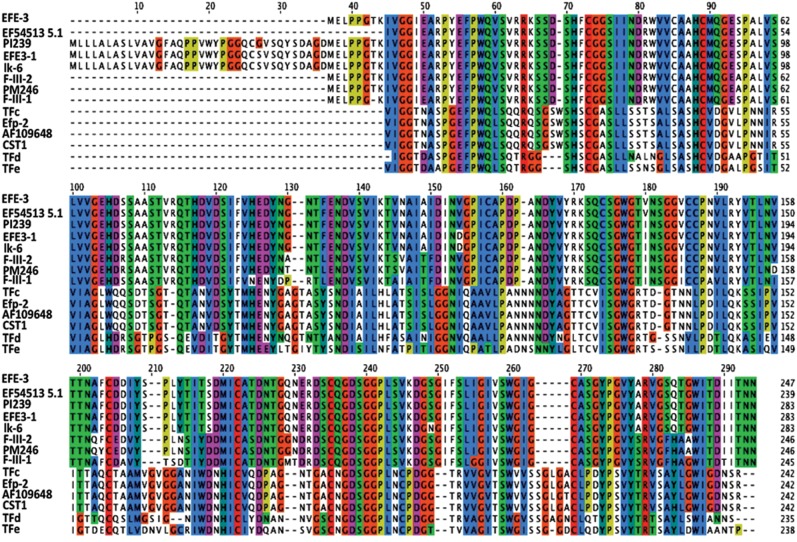
Multiple sequence alignment of CST1 amino acid sequences. The reported lumbrokinase amino acid sequences were from GenBank. Identical residues are shadowed with the same color.

**Figure 4:**
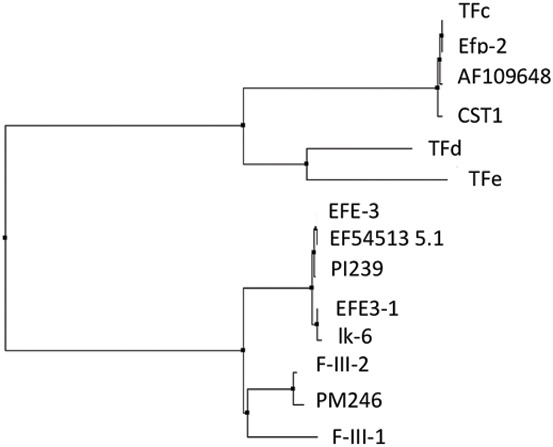
Phylogenetic tree analysis of CST1 amino acid sequences. Phylogenetic analyses was performed with ClustalW software using the Neighbor joining method.

**Figure 5:**
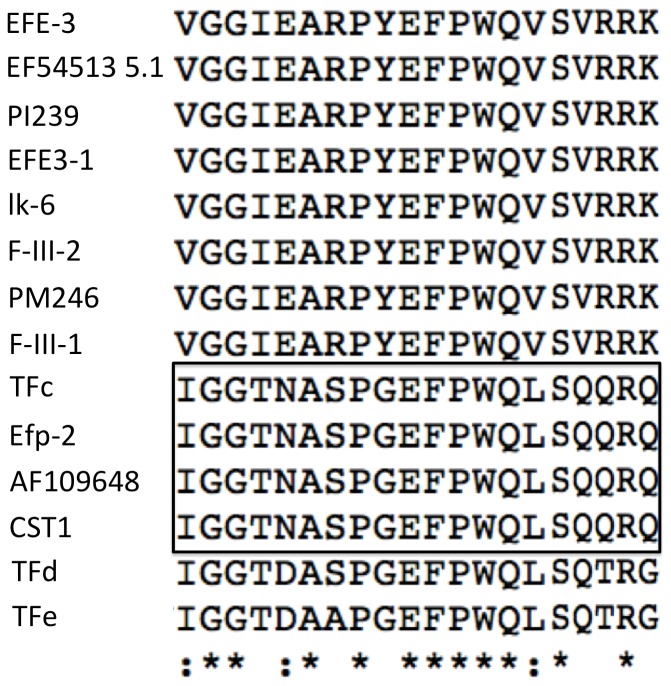
The N-terminal sequences of lumbrokinases. Identical amino acids are indicated by an asterisk (*). Boxed region represents identical N-terminal amino acids.

Alignment of multiple lumbrokinase sequences revealed that the sequences of lumbrokinases are highly conserved (Fig. 3). The deduced amino acid alignment showed that CST1is most closely related (99% sequence identity) to the gene, AF109648, from *L. bimastus*, as well as TFc (EU167735.1), and Efp-2 (DQ836918.1) from *E. fetida*. The amino acid sequence of CST1 exhibited less than 35% identity with other translated earthworm lumbrokinase cDNA sequences (Table 1). The phylogenetic tree constructed for CST1 on the basis of amino acid alignment also showed that this enzyme is more related to TFc, Efp2, and AF109648 (Fig. 4). They all share a more recent common ancestor with each other than they do with F-III-1, F-III-2, EFE-3, EFE3-1 and lk6 found in *L. rubelllus*, or PI239 and PM246 found in *L. bimastus*. N-terminal amino acid comparison showed that CST1 shares a similar sequence with other lumbrokinases. It has the identical sequence as TFc, Efp-2 and AF109648 (Fig. 5).

**Table 1:** CST1 amino acids identities with other lumbrokinases.

Earthworm	Lumbronkinase	GenBank No.	Identity
*E. fetida*	TFc	EU167735.1	99%
	Efp-2	DQ836918.1	99%
	TFd	EU167736.1	69%
	TFe	EU167737.1	65%
		EF545135.1	34%
*L. bimastus*		AF109648	99%
	PI239	AF433650	33%
	PM246	AY178854.1	33%
*L. rubelllus*	F-III-2	AB045720	34%
	EFE-3	AY438622	33%
	EFE3-1	U25643	33%
	F-III-1	AB045719	35%
	lk-6	AF304199	33%

**Figure 6:**
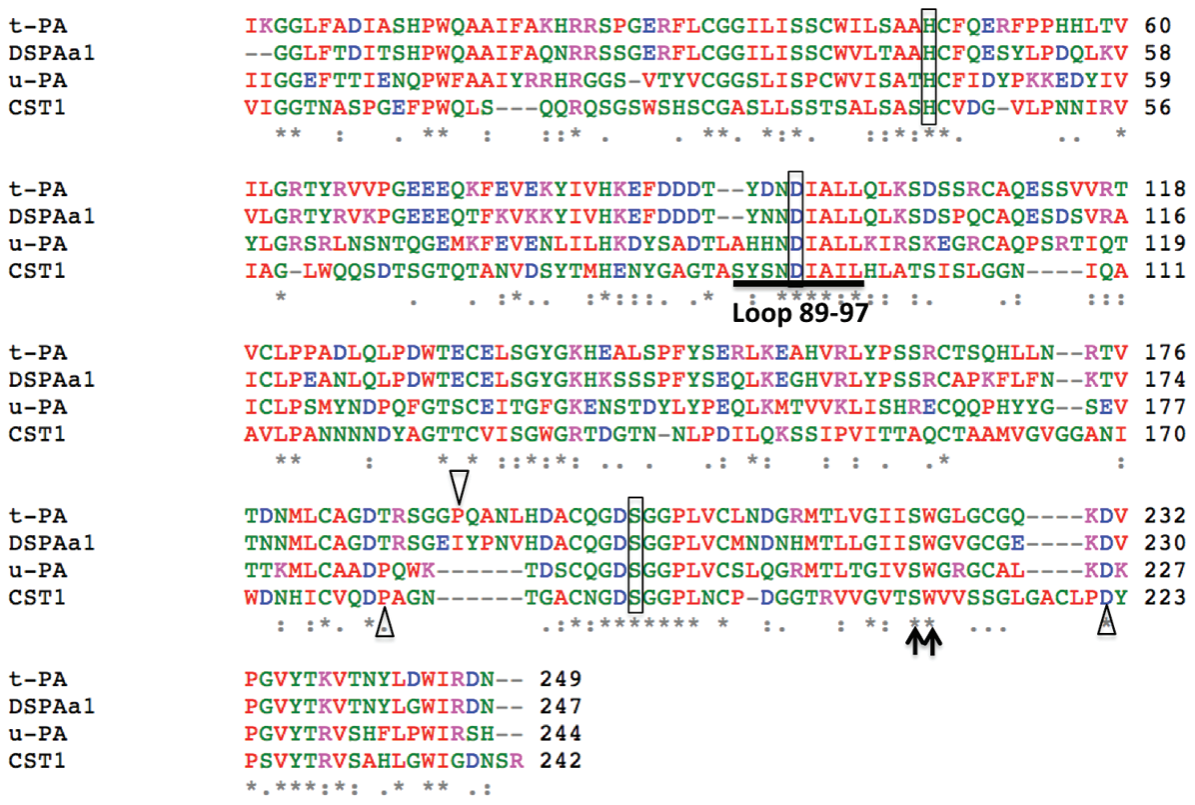
Alignment of proteolytic domain sequences. CST1, human t-PA, u-PA and vampire bat plasminogen α1 (DSPAα1) amino acid domains were analyzed. Identical amino acids are indicated by an asterisk (*). Boxed region represents the catalytic triad amino acid: histidine (H), aspartate (D) and serine (S). Subsites S1 and S2 are indicated by an arrow (↑). Substrate recognition amino acids are indicated by an empty Triangle (Δ). The specificity pocket and loop are underlined.

tPA, urokinase-type plasminogen activator (uPA) and vampire bat (*Desmodus rotundus*) plasminogen activator (DSPAα1) are highly specific serine proteases which catalyze the conversion of plasminogen to plasmin for breaking down fibrin or to specifically target fibrin [25]. When CST1 was aligned with the serine protease domain of tPA, uPA and DSPAα1, amino acid similarities were low (from 29% to 32%) (Fig. 6). However, they share common features. CST1 has the conserved catalytic triad, histidine (H^44^), aspartate (D^93^), and serine (S^191^), found in the active sites of protease enzymes. CST1 has a high similarity loop and specific pocket (Ser^89^ to Leu^97^) similar to tPA, uPA and DSPAα1. CST1 also contains the amino acids, Pro^180^ and Asp^222^, which are very important substrate recognition sites for tPA, uPA and DSPAα1. Ser^209^ and Trp^210^ of CST1 match the S1 and S2 subsites of tPA, uPA and DSPAα1.

### CST1 expression and purification, SDS-PAGE and Western Blot analysis

**Figure 7:**
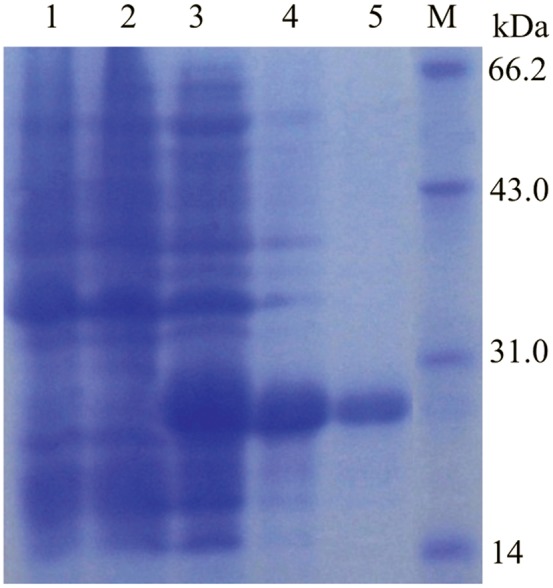
SDS-PAGE analysis of rCST1. Lane 1: Crude lysate of *E. coli* BL21 transformed with empty plasmid; Lane 2: Uninduced sample; Lane 3: Total soluble proteins extracted from induced cells; Lane 4: Solubilized CST1 from inclusion body; Lane 5: Purified, refolded CST1 with 6-His column chromatograph; M: protein molecular marker.

The *CST1* ORF fragment was cloned into the expression vector, pET28(+), with a 6xHis tag driven by a T7 promoter. Most of the rCST1 was found in the *E. coli* insoluble inclusion bodies (data now shown) which is consistent with other recombinant lumbrokinase proteins expressed in *E. coli* BL21(DE3)cells [19], [26]. After ProBond™ column (Invitrogen) purification, purity of the concentrated CST1 protein was evaluated by 12% SDS–PAGE analysis (Fig. 7). Purified, refolded rCST1 shows sharp band with a MW about 25 kDa, which corresponds to the predicted molecular weight of CST1. rCST1 was approximately 95% pure as analyzed by 12% SDS-PAGE. About 50% CST1 protein was recovered as determined by densitometry (UVP Lab Works 3.0) which equates to a yield of 170 mg/L.

**Figure 8:**
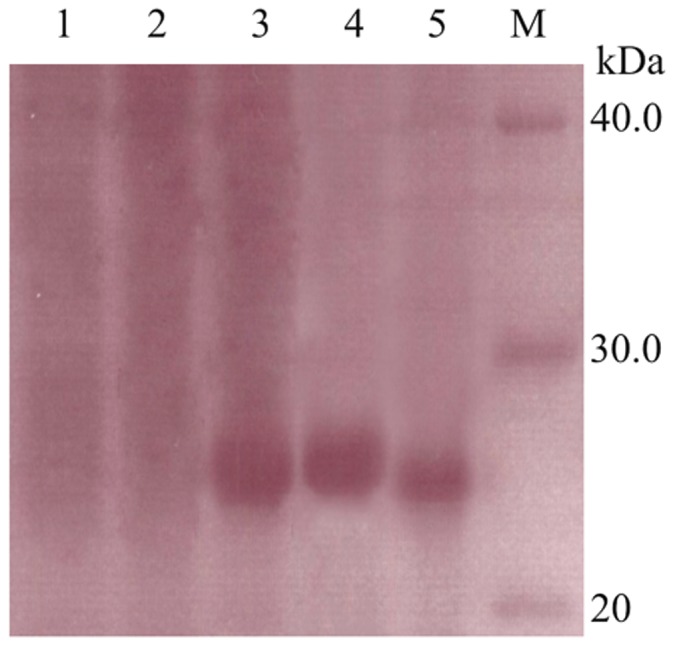
Western Blot analysis of rCST1. Lane 1: Crude lysate sample before IPTG induction; Lane 2: Supernatant from inclusion body in induced cells; Lane 3: Unpurified refolded CST1 extract from inclusion body; Lane 4: Purified refolded CST1; Lane 5, Standard lumbrokinases; M: protein molecular marker.

The antibody developed against rCST1 was detected by ELISA (data not shown). From one week after the fourth immunization, ELISA showed a significantly high antibody response (data not shown) indicating the enhanced immunogenicity of rCST1. Western blot analysis (Fig. 8) revealed an immune response of the mouse antibody with rCST1indicating that the expressed, purified rCST1 has the correct conformation rCST1 was almost undetectable in the inclusion body supernatant indicating that most of rCST1 was expressed as inclusion protein. As expected, a molecular weight of 25 kDa band was detected. A band was also detected in the positive control, a standard lumbrokinase product (Fig. 8, lane 5).

### Fibrinolytic activity assay

**Figure 9:**
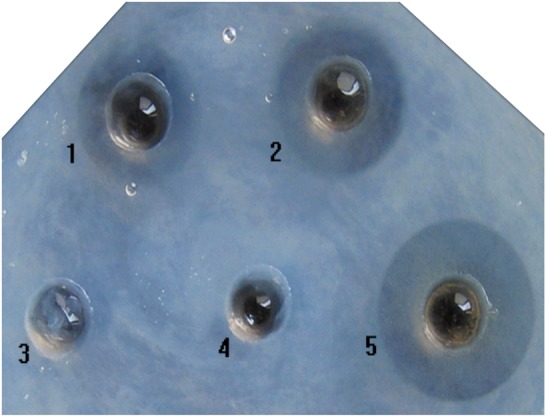
Fibrin plate assay. Well 1: Unpurified refolded rCST1; Well 2: Purified refolded CST1; Well 3: Crude lysate sample before IPTG induction; Well 4: Non-refolded solubilized CST1 extract from inclusion body; Well 5: Standard lumbrokinases (1200 u/mg).

The fibrinolytic activity of the refolded rCST1 was revealed using the fibrin plate assay (Fig. 9). Both purified and non-purified refolded rCST1 showed a half-transparent lytic area on the fibrin plate, indicating that fibrin had been degraded into soluble peptides. The lytic area of rCST1 was smaller than the standard lumbrokinase, suggesting that the fibrinolytic activity of the rCST1 is less than the standard lumbrokinase. The mean (n = 3) diameter of the lytic areas of rCST1 and the commercial lumbrokinase product (1200 u/mg) were 1.14 cm and 1.50 cm, respectively. The fibrinolytic activity of rCST1 was calculated as 912 u/mg. Thirty six amino acid residues and the HIS tags at the N-terminal of rCST1from the vector pET28a(+) were not cleaved. Those residues did not seem to affect rCST fibrinolytic activity.

### Blood-clot lysis activity assay

**Figure 10:**
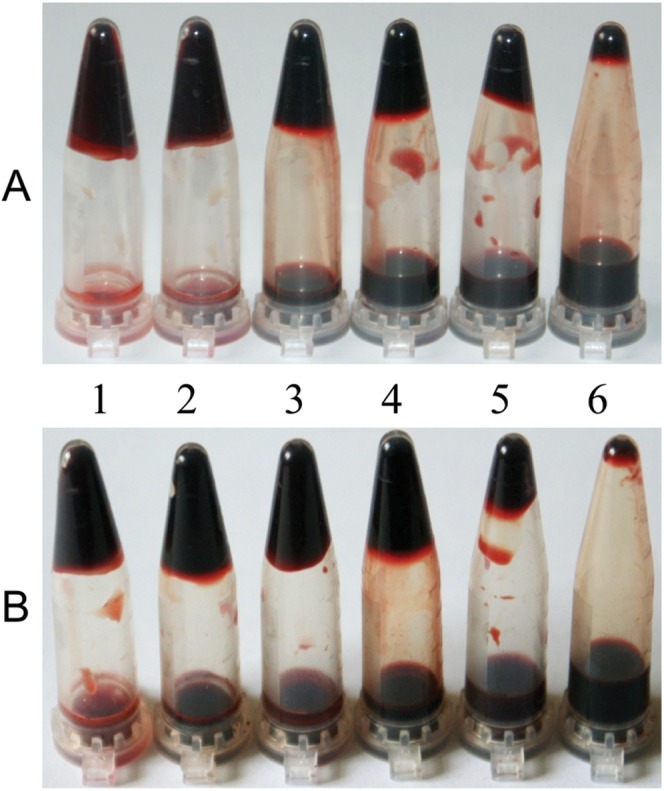
Blood-clot lysis assay. The purified refolded rCST1 (A) and standard lumbrokinase (1200 u/mg) (B). Tube no. 1: negative control 1×PBS; Tube no 2 to 6: sample with concentrations of 5, 10, 20, 40 and 80 mg/mL.

**Figure 11:**
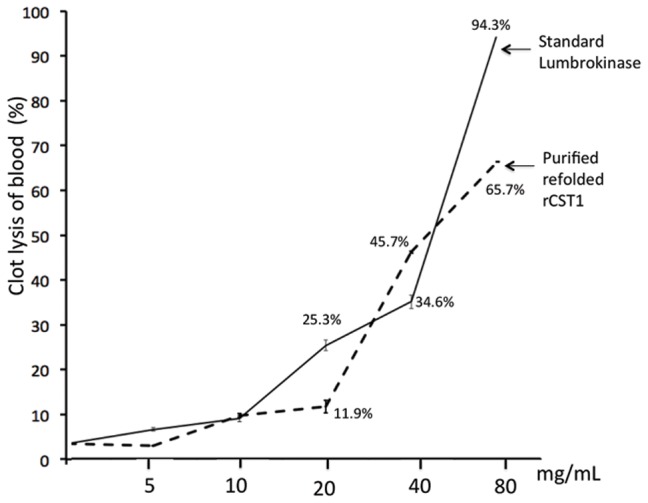
Percent blood-clot lysis. Purified refolded rCST1 and standard lumbrokinase (1200 u/mg) clot lysis was monitored by incubating a tube of clotted blood with different concentrations (0, 5, 10, 20, 40 and 80 mg/mL) of lumbrokinase at 37°C for 2 h. Weight difference between untreated and treated samples as expressed as % of clot lysis. The data shown are means of three replicates. Values are means ± SD (n = 3). Regressive equation of standard lumbrokinase: Y = 0.008x-0.3068; correlation coefficient R^2^ = 0.97482.

Visual blood clot lysis is shown in Figure 10. When 1×PBS (negative control) was added to the blood clot, no obvious lysis was observed. Both standard lumbrokinase and rCST1 increased the percentage of clot lysis with increasing protein concentration. Highest clot lysis (94.3% and 65.7%) was observed with the addition of 80 mg/mL of standard lumbrokinase or rCST1, respectively. Upon the addition of 40 mg/mL of samples, the clot lysis percentage of rCST1 (45.7%), however, was significantly higher than that of standard lumbrokinase (34.7%, p<0.001) (Fig. 11). No evidence of clot lysis was observed when blood samples were treated with the non-refolded inclusion body containing rCST1. These data indicate that at certain concentrations (40 mg/mL), rCST1 is more reactive with the blood clot. More research is needed, however, to clarify this dosage response.

## Discussion

**Figure 12:**
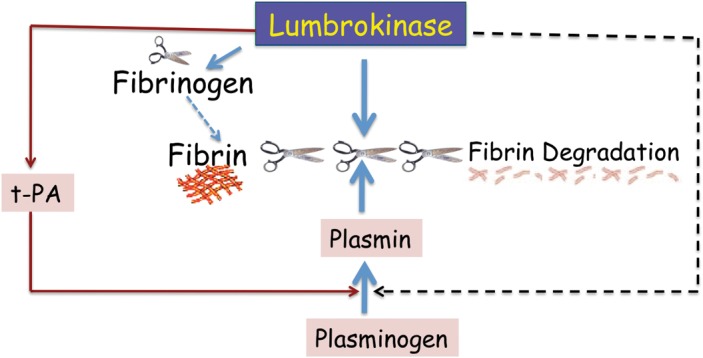
Lumbrokinase Mechanism of Action. Dissolves fibrin and fibrinogen directly; Converts plasminogen to plasmin; Enhances endogenous tissue plasminogen activator (t-PA).

Lumbrokinase can dissolve fibrinogen and fibrin directly. It also can convert plasminogen to plasmin and increase endogenous t-PA activity to dissolve fibrin clots (Fig. 12). Lumbrokinases dissolve blood clots and are becoming an effective tool for the treatment and prevention of cardiovascular disease. Current lumbrokinase products contain multiple components extracted from earthworm which causes nausea and vomiting. In the present study, a full-length lumbrokinase gene, CST1, was cloned from total RNA of the earthworm, *E. fetida,* by RT-PCR amplification. CST1 contains 19 asparagine (N) and 13 aspartic acid (D). It only has a single lysine (K). Sequence alignment of CST1with other lumbrokinases indicates (Fig. 3) that it belongs to the family of serine proteases which all have more asparagine (N) and aspartic acid (D) residues and less lysine (K) [27]. Disparate amino sequences may have evolved in response to the food digested by the earthworms [21], [27].

A comparison of the conserved N-terminal sequences (Fig. 5) of CST1 with other lumbrokinases revealed the critical physiological role of this portion of the protein to the hydrolysis of fibrin and other proteins [20]. CST1 possesses the conserved, catalytic triad (Fig. 6) known to critical for the hydrolysis of targeted proteins. CST1 shares a conserved loop, as well as specific pocket and substrate recognition sites with tPA, uPA and DSPAα1. These sites play a coordinated role in the catalysis and cleavage of peptide bonds. The structural features conserved in CST1 support its strong fibrinolytic activity. The presence of the conserved sites in CST may explain why lumbrokinases can convert plasminogen to plasmin (like tPA) to dissolve fibrin clots [28]. Similar to DSPAα1 [29], lumbrokinases also have a significant specificity for fibrin, thus reducing excessive bleeding.

Western blot assay (Fig. 8) did not detect inclusion body rCST, indicating that most of the rCST1 was expressed as inclusion protein. rCST antibody reacted with the standard lumbrokinase product suggesting that standard lumbrokinase product, extracted from the intestinal tract or tissue fluid of earthworms may have a specific component with a similar protein structure to CST1.

The fibrin plate (Fig. 9) and blood-clot lysis assays (Fig. 10) revealed that purified rCST1 has fibrinolytic activity. It also indicates that rCST1 is very stable as the fibrinolytic activity was preserved despite being subjected to several purification steps. Inclusion body rCST1 did not show lytic activity which means that the refolding process is necessary for rCST1 activity. Similar findings have been reported for other lumbrokinase expressed in *E.coli* [26].

Both the fibrin plate and blood-clot lysis assays indicated that the activity of rCST1 is lower than the standard lumbrokinase product, suggesting that the standard lumbrokinase product may contain several lumbrokinases and other proteases. Multiple components may exhibit synergistic activity where the different components work together to dissolve fibrin and show stronger fibrinolytic activity. Another reason for the lower fibrinolytic activity of CST1 may be due to the fact that it was expressed as an inclusion body in *E. coli,* thus reducing its enzymatic activity after the denaturation and refolding process. Wu et al. [30] reported that eight trypsin-like isozymes isolated from *E. fetida* are glycosylated. In eukaryotic cells, glycans are very important for proper protein folding, maintenance of protein conformation and solubility, and stability and mediation of biological activity [31]. The CST1 gene, cloned from *E. fetida* may be a glycoprotein. *E. coli* is not always the optimum organism for expressing eukaryotic glycoproteins since it lacks post-translational modification and protein folding machinery [32]. CST1 might loss some fibrinolytic function and activity without proper glycosylation. It's worth noting that if the serum was not removed after the blood clot formation in the *in vitro* clot fibrinolytic experiment, no lysis was observed with either the standard lumbrokinase or rCST1. The basis for this inhibition is unclear and warrants further investigation.

Since the isolation and purification of a single lumbrokinase has been unsuccessful from raw extracts obtained from earthworm, our work provides a promising foundation for the genetic engineering of single lumbrokinase protein for future pharmaceutical usage. We have successfully cloned and expressed a novel lumbrokinase gene, CST1, in *E.* coli, and demonstrated its fibrinolytic activity. CST1 displays both strong blood-clot lysis and fibrin degradation activity. CST1, however, was expressed as an inclusion body in *E.coli*. Purification and renaturation of recombinant proteins in *E.coli* is still a tedious process. Additional studies are in progress to optimize CST1 expression, improve its yield, and enhance its fibrinolytic activity.
